# Synthesis, Properties and Applications of Polymeric Nanomaterials

**DOI:** 10.3390/nano12244385

**Published:** 2022-12-09

**Authors:** Massimiliano Perduca

**Affiliations:** Biocrystallography and Nanostructure Laboratory, Department of Biotechnology, University of Verona, Strada Le Grazie 15, 37134 Verona, Italy; massimiliano.perduca@univr.it

The term “polymeric nanomaterials” is commonly used for all polymer-based nanomaterials, but it is mainly applied to nanospheres and nanocapsules. Generally defined as colloidal systems, polymeric nanoparticles have attracted considerable interest all over the world due to their unique properties and their extensive applications in various fields, such as drug delivery systems, biosensors, catalysts, nanocomposites, agriculture, environment, etc. Based on their origin, from either synthetic or natural polymers, and on their in vivo behavior, polymeric nanomaterials can be classified as biodegradable or non-biodegradable. These classifications are used to encapsulate a wide variety of compounds, ranging from hydrophobic drugs, water-insoluble chemicals, metals, nutraceutics, bioactive compounds, proteins, and nucleic acids, in order to describe their physicochemical properties and mechanisms of interaction with living cells.

This Special Issue, focused on polymeric nanomaterials, reports the latest advances in the synthesis, properties, and applications of this useful and versatile class of nanocomposites. These compounds can be used in drug delivery for the treatment of cancer cells, providing a platform for the co-delivery of nucleic acids and hydrophobic drugs [1]. Another area where such nanopolymers can fruitfully be applied is bone regeneration for maxillofacial reconstructive surgery; new nanomaterials based on hydroxyapatite and poly-l-lactide acid have been shown to be able to recover critical defects in rat mandibles [2], paving the way to their use in humans. Biocompatible and biodegradable polymers can also be used to enhance the biocompatibility and the consequent cellular uptake of different classes of nanocompounds, where their poor ability to enter cells potentially limits their application [3]. Such novel nanomaterials have acquired new features without any loss of efficacy for the encapsulated nanoparticles. Another area of the medical sciences where polymeric nanomaterials can be effective is the development of new antibacterial compounds for the production of protection devices or for use during surgery to prevent infectious diseases [4]. Polymers are also having a strong impact in the development of new technologies and the use of new nanopolymeric matrices in 3D printing, which can have positive effects on the production of new optical elements, with enhanced fluorescent emission [5]. Moreover, another aspect considered in this Special Issue concerns the optimization of the production of polymeric nanomaterials towards a green chemistry approach, starting from biodegradable and sustainable materials and involving the use of low-toxicity solvents to obtain environmentally friendly nanomaterials [6].

In summary, the development of polymeric nanomaterials is approached in this Special Issue from several different points of view, and I hope that readers will find here new ideas that are useful for the development of their future research.
